# Bubble-Induced Cave Collapse

**DOI:** 10.1371/journal.pone.0122349

**Published:** 2015-04-07

**Authors:** Lakshika Girihagama, Doron Nof, Cathrine Hancock

**Affiliations:** 1 Geophysical Fluid Dynamics Institute, The Florida State University, Tallahassee, Florida, United States of America; 2 Department of Earth, Ocean, and Atmospheric Science Department, The Florida State University, Tallahassee, Florida, United States of America; Coastal Carolina University, UNITED STATES

## Abstract

Conventional wisdom among cave divers is that submerged caves in aquifers, such as in Florida or the Yucatan, are *unstable* due to their ever-growing size from limestone dissolution in water. Cave divers occasionally noted partial cave collapses occurring while they were in the cave, attributing this to their unintentional (and frowned upon) physical contact with the cave walls or the aforementioned “natural” instability of the cave. Here, we suggest that these cave collapses do not necessarily result from cave instability or contacts with walls, but rather from divers bubbles rising to the ceiling and reducing the buoyancy acting on isolated ceiling rocks. Using familiar theories for the strength of flat and arched (un-cracked) beams, we first show that the flat ceiling of a submerged limestone cave can have a horizontal expanse of 63 meters. This is much broader than that of most submerged Florida caves (~ 10 m). Similarly, we show that an arched cave roof can have a still larger expanse of 240 meters, again implying that Florida caves are structurally stable. Using familiar bubble dynamics, fluid dynamics of bubble-induced flows, and accustomed diving practices, we show that a group of 1-3 divers submerged below a loosely connected ceiling rock will quickly trigger it to fall causing a “collapse”. We then present a set of qualitative laboratory experiments illustrating such a collapse in a circular laboratory cave (i.e., a cave with a circular cross section), with concave and convex ceilings. In these experiments, a metal ball represented the rock (attached to the cave ceiling with a magnet), and the bubbles were produced using a syringe located at the cave floor.

## Introduction

**Fig 1:**
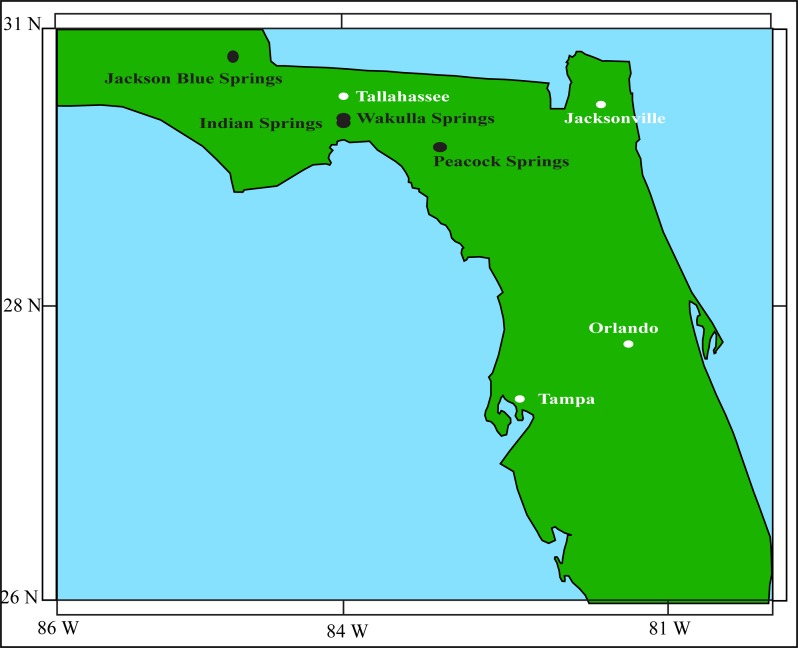
Florida map. A map showing the approximate locations of the Jackson Blue Springs, Indian springs, Wakulla Springs, and the Peacock Springs. See text for a discussion of these springs. Information taken from Esri, DeLorme, USGS, NPS | Esri, GEBCO, DeLorme, NaturalVue | Esri, GEBCO, IHO-IOC GEBCO, DeLorme, NGS.

**Fig 2:**
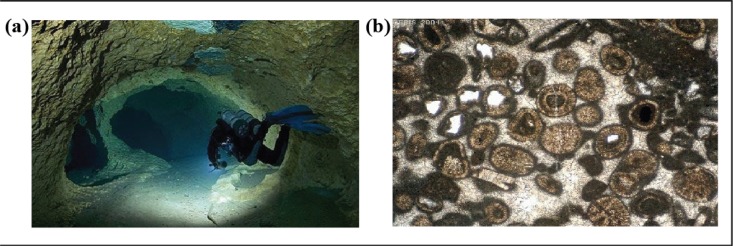
The two domains of a typical “karst” aquifer. The so-called matrix (right) is composed of sand, clay and broken limestone (individual particles are roughly one millimeter in diameter) and looks essentially like a sponge filled with water. This part of the aquifer is common in most of the world (image credit: D J Waters and the Department of Earth Sciences, University of Oxford). On the left is a typical Florida conduit (a few meters in diameter) leading to the Florida springs (courtesy of Jill Heinrich—IntoThePlanet.com). The Florida “karst” aquifer consists of a mixture of the two.

In contrast to most aquifers in the world (consisting of a continuous and relatively uniform porous media), the Florida aquifer is a mixture of tunnels and a continuous matrix, usually referred to as a “karst” aquifer (Figs 1 and 2). Such an aquifer looks like “cheese” in the sense that it contains both tunnels (a few meters in diameters as shown in the left panel of Fig 2) and a porous limestone medium whose particles have a scale of a millimeter or less (Fig 2, right panel). (The name “karst” originates from the German name for “Kras”, a region in Slovenia where the first scientific research of karst topography was made.) Karst caves are formed by groundwater gradually dissolving the surrounding limestone such that their size is constantly growing.

### a) Cave collapse in Karst aquifers

There are at least two kinds of (submerged) underground cave collapse processes in a karst aquifer. The first one is a “major collapse” where, due to limestone’s continuous dissolution in water, the cave’s ceiling becomes too thin to support the ever-growing cave and it collapses to form a sinkhole. We shall speak about this case in Section 2, where it will be shown that, while this kind of collapse is common in some isolated parts of Florida, most Florida caves are too small for such a collapse to occur. The second (less major) kind of submerged cave collapse is where a single or a group of ceiling rocks fall and either partially or completely block the cave (to cave-divers penetration). This is a much smaller collapse process that does not necessarily involve the dissolution of limestone and subsequent formation of a sinkhole. It is this collapse process that we focus on in this paper. One example of such a collapse is the 2011 collapse in Jackson Blue, Marianna, Florida, where a 100 tons ceiling rock fell to the cavern floor. Though this rock is probably too large to be associated with the processes we propose later, it does belong to the category mentioned above. Another is a very recent collapse (2013) where a one or two-ton ceiling rock fell and blocked one of the Peacock Spring exits/entrances (See Fig 1).

There is actually a third kind of collapse involving *partially* submerged caves. When ground water is excessively pumped out of the aquifer (for agricultural use), the water table is sometimes forced below the caves’ ceiling causing a loss of roof- supporting buoyancy. These caves, now only partially submerged, often collapse to form a sinkhole. There is anecdotal evidence for a dramatic increase in formation rate of such sinkholes after deep freezes, when more ground water is pumped out to water the trees and save the oranges from freezing. However, since this kind of collapse involves partially submerged caves, it is of no interest to the current study.

### b) Accidents

As there is no sunlight in caves, and due to the filtering ability of porous limestone, water in these caves is the clearest in the world. In part, for this reason, films like “Airport 77” have been shot in Wakulla Springs. This has also attracted specially trained cave divers, driven primarily by curiosity, to penetrate long distances into these caves with some excursions exceeding a few kilometers and lasting for many hours, if not days. Like “base jumping” (a branch of skydiving), this sport is considered by many as an “extreme” sport (http://en.wikipedia.org/wiki/Cave_diving). It is hard to come by worldwide statistics of cave diving accidents, but in Florida alone there have been several hundred fatalities during the past 50 years. Most of them were due to lack of adequate training and/or inappropriate gear.

With the exception of one well-known case, where the cave literally collapsed while divers were in it, all of these accidents were due to diver error as opposed to environmental causes. It has been suggested recently, that the exceptional case in question of no-diver-fault was probably induced by the bubbles released by the divers, which caused *resonance* in the cave, leading to a collapse [1–2]. This implies the accident was due to divers actions, though, at the time, it was unknown this could cause a collapse.

Although this is the only documented case of an accident caused by a cave collapse, there are many anecdotal collapses where rocks fell from the ceiling while cave divers were going through the cave. The typical anecdotal explanation given on most cave diving forums is that caves are inherently “unstable” and this is why rocks fall from the ceiling from time-to-time. While this explanation is psychologically comforting to cave divers because it places no responsibility on the divers themselves, we shall show here that it is probably incorrect in most cases. We shall argue that, *even without resonance*, the gas released by the divers can cause a collapse (by lowering the buoyancy that forces the ceiling upward).

### c) Stability and the loss of buoyancy

As mentioned, some caves may indeed be unstable, as limestone dissolution allows them to grow too large for the ceiling, which acts like a beam, to support their weight and the weight above it. Simple calculations presented in Section 2 suggest however, that for most caves this is not the case. In other words, Florida caves can grow to sizes much larger than their actual sizes today. Yet, since large rocks do occasionally fall from the ceiling, this suggests a process unrelated to cave stability. We shall show in section 3, that these falling rocks are probably a direct result of buoyancy reduction on isolated ceiling rocks, due to divers bubbles. (This reduced buoyancy acts like added weight to rocks hanging from the ceiling.) For example, we shall see that three divers exiting a slanted cave slowly (15 meters per minute) through a 75 meters long and 5 meters in diameter chimney extending from 30 to 10 meters will exert a buoyancy reduction of as much as 500 kilograms. (Strictly speaking, buoyancy is measured in Newtons but, for simplicity, we shall use Kilograms, which is the unit usually used to measure weight).

## Ceiling Instability of Slowly Growing Caves

**Fig 3:**
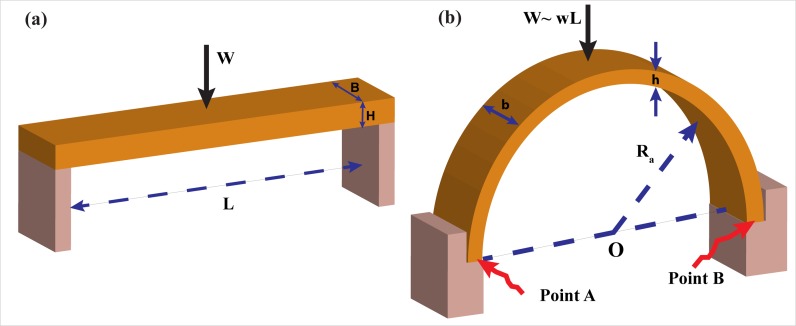
3-D schematic of a flat and concave roof. (a) The width and height of the flat beam is *B* and *H*, respectively. The beam rests loosely on the sidewalls, where *L* and *W* are the beam span and self-weight in water, respectively. (b) The width and height of the concave roof are *b* and *h*, respectively. Weight of the beam in water is *W*, and the radius of the semicircular arch is *R*_*a*_. The lower case w represents the weight of a unit length.

In this section we will discuss the stability of two kinds of caves shown in Fig 3, one with a flat roof and the other with a more realistic (and more stable) concave roof. We shall see that both cave configurations are very *stable* when using standard dimensions of Florida and Yucatan caves. In fact, these caves do not become unstable until their size exceeds that of Florida and Yucatan caves by almost an order of magnitude.

### a) Flat roof

**Fig 4:**
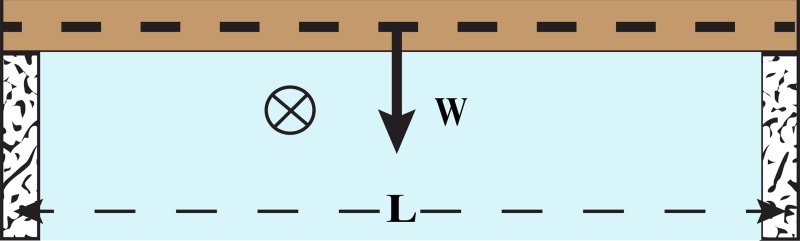
A rectangular beam. The cross-sectional area, *BH* (not shown, *B* and *H* are beam width and height, respectively), represents the roof covering the cave tunnel. The beam rests loosely on the sidewalls, where *L* and *W* are the beam span and self-weight, respectively. Water flow in the cave is into the paper, denoted by ^⊗^.

Consider the situation in Fig 3a, shown as a 2D image in Fig 4 for ease of understanding. The rectangular rock, with a cross-sectional area BH (where B and H are rock width and height, respectively), represents the roof covering the cave tunnel. The cave span next to the ceiling is L, where the rectangular rock, loosely resting on the sidewalls, is taken to be slightly longer than L. Note that variables are defined in the text.

Using standard and familiar rock mechanics (see e.g. [3]) we calculate that the maximum *shear* stress (τ), occurring at the centerline of the beam, is

τ = 3W/ 4BH= 3ρ′ gL/ 4 (1)

where *ρ*′ is the *submerged* rock density (*ρ*′ = *ρ*_R—_ *ρ*_*W*_, where *ρ*_*W*_ and *ρ*_R_ are the densities of water and the rock in air, respectively), *g* the gravitational acceleration, and *W* the weight of the flat beam in water. We shall see shortly that this vertical shear stress is negligible in regards to the stability process we are now addressing.

As is normally the case, the beam is compressed in its top part and stretched in the lower part. The maximum horizontal *tensile* stress, also in the middle of the beam, is σ = *MH* / 2*I* where *M* = *WL* / 4 *and I* = *BH* ^3^ / 12. Here, *M* is the maximum bending moment and *I* is the moment of inertia. These ultimately give,

$$\sigma \;=\;3\rho \prime \;gL^{2}/\;2H$$ (2)

where σ is the maximum failure tensile stress. Note that, for L >> H (our present case, but not necessarily the norm), the tensile stress is much larger than the shear stress. In contrast, when H > L, the shear stress is the largest. We shall see later that, in the case of a shear failure, the critical condition is independent of depth due to equal growth rates of thickness and weight (see Eq 1). Next, we take the maximum shear strength of limestone as 35 MPa (1 MPa = 10^6^ Pa). This follows Mohr-Coulomb failure criterion, stating that the shear strength is half the compressive strength of a material. According to [4], limestone’s compressive strength varies from 70–210 MPa. We take the conservative approach and use the lower bound, 70 MPa, for our calculations. We find that the maximum span of the cave is incredibly large, about 2800 meters, clearly beyond the range of Florida caves.

[5] and [6] suggest the failure *tensile* stress is in the vicinity of 5 MPa, indicating that the limestone is slightly stronger than concrete. Taking the beam thickness (i.e., the cave ceiling depth below ground) H to be 20 m, the limestone critical strength to be 5 MPa and *ρ*′ to be 1.7 *gr/cm* ^*3*^ (corresponding to a density in air of 2.7 *gr/cm* ^*3*^), we find using (Eq 2) that the beam will break (i.e., the cave will collapse) when it reaches a span of ~ 63 m. This is much broader than most of Florida’s submerged caves, which are usually no more than 10–20 meters broad, demonstrating that most Florida caves are *stable* to general failure. The presence of cracks will, of course, reduce the above stability criteria; however, it is very difficult to estimate what will happen in such a case. Assuming the critical cracks are vertical and occupy half of the roof thickness (i.e., taking H = 10 m instead of 20 m), we find the critical cave width to be 45 m (instead of 63), which is still double the size of most Florida caves. Note that a cave roof with the above limestone strength will need to be *less than 50 cm* thick in order to be unstable at a 10 m span—an extremely unusual situation for most Florida caves. Also, note that the effect of layering on the beam strength needs detailed analysis and is beyond the scope of this study.

### b) Concave roof

The arched ceiling case is a much more stable configuration than the previously considered flat roof case, due to minimal tensile stress. With an arched ceiling, most of the stress is of *compression* form, which stabilizes the cave since limestone is six times more resistant to compression stress than it is to tensile stress. (This idea of increased arched stability was known even to the Romans, who used arched beams throughout their structures. Other cultures were probably also familiar with this concept.)

**Fig 5:**
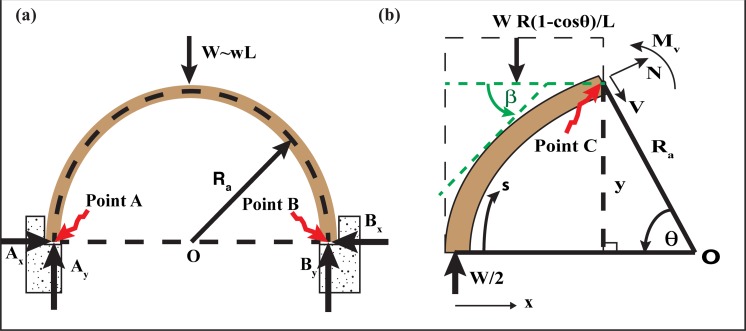
Arched roof. (a) A symmetrical semicircular two-hinged arch with a rectangular cross section (width *b*, thickness *h*). The vertical and horizontal forces at points A and B are *A*_*y*_, *B*_*y*_, *A*_*x*_, and *B*_*x*_ respectively. Weight of the beam in water is *W*, radius of the semicircular arch is *R*_*a*_, span of the arch is *L (= 2R*_*a*_ *)* and lower case w represents the weight of a unit length. (b) A section of the semi-circular arch given in Fig 4a. *N* and *V* are the normal thrust and shear forces, respectively, at any point on the arch, and *θ* is the angle *V* makes with the horizontal. *β* is the angle of the tangent at any point on the arch to the horizontal axis.

To examine this case, consider a symmetrical two-hinged arch (Figs 3b and 5a) with a rectangular cross section (width *b*, thickness *h*). Based on the symmetry of the structure, one easily finds that the vertical reactions at the points A and B, are *A*_*y*_ = *B*_*y*_ = *W* / 2, where *W* is the arch weight in water. Next, we denote the unknown horizontal reaction *F* at points A and B by |*A*_*x*_| = |*B*_*x*_| = *F*.

Using familiar mechanical principles, ([7], (see (his) equations (4.5), (4.6) and (4.8a)) showed that this horizontal force (see Fig 5) is,

$$F=\left(\int_{A}^{B}M_{\upsilon }y\;ds\;/\;EI-\int_{A}^{B}N\;\cos\beta \;dx/EA_{c}\right)/\left(\int_{A}^{B}y^{2}\;ds\;/\;EI+\int_{A}^{B}\;\cos^{2}\beta \;dx/EA_{c}\right)$$ (3)

where *E* is the modulus of elasticity, *A*_*C*_ the cross section area (= *bh*), *I* the moment of inertia (*bh* ^*3*^ */12*), *β* the angle of the tangent at any point on the arch to the horizontal axis, N the normal thrust any point on the arch, *M*_*υ*_ the bending moment (at any section) due to the load alone, *y* the vertical distance from a point on the arch to the support points (A or B) (*y = R*_*a*_ *sinθ*,), *R*_*a*_ the radius of the semicircular arch, *θ* the angle of the section of the arch makes with the horizontal, s the centerline length of the arched beam, and *x* the distance along the horizontal axis. Note that (Eq 3) involves three integration elements, *x*, *y* and *s*.

It can be shown that the contribution from the normal force N is small compared to the actual load [7], so that the second term in the numerator becomes negligible. Further, we assume that the axial rigidity (*EA*_*C*_) of the arch is high, thus the second term in the denominator is also negligible. Consequently, (Eq 3) reduces to

$$F=\int_{A}^{B}M_{\upsilon }yds/\int_{A}^{B}y^{2}ds$$ (4)

The weight–induced moment function *M*_*υ*_ is now obtained by taking $\sum M_{\sec tion}=0$ about point C (Fig 5b), giving *M*_*υ*_ = (w / 4)R_*a*_ sin^2^ *θ*, where, *W = πR*_*a*_ *bhρ*′ g.

Substituting this into (Eq 4) yields,

F = 2W / 3π (5)

Note that we have horizontal and vertical forces at points A and B. First, let’s calculate the bending moment (M) at any point on the beam. By taking $\sum M_{\sec tion}=0$ about any point on the beam (equivalent to point C (Fig 5b) but with known *A*_*x*_ and *A*_*y*_ at point A) and using (Eq 5), we find the total bending moment (*M*) at any angle (*θ*) to be

$$M_{}=\;\left(w\;/\;4\right)R_{a}sin^{2}\theta –\;\left(2W/\;3\pi \right)R_{a}sin\;\theta$$ (6)

Noting that the maximum moment function (*M*_*max*_) occurs when *sin θ* = 1, we get

$$M_{max}=\;\left(W/\;4\right)R_{a}–\;\left(2W/\;3\pi \right)R_{a}$$ (7)

Further noting that, by definition, the maximum stress (*σ*_*max*_) is given by

$$\sigma_{max}{=}_{}M_{max}y/I$$ (8)

we get using (Eq 7),

$$\sigma_{\max}=(3\pi -4)R_{a}^{2}\rho 'g/h$$ (9)

**Fig 6:**
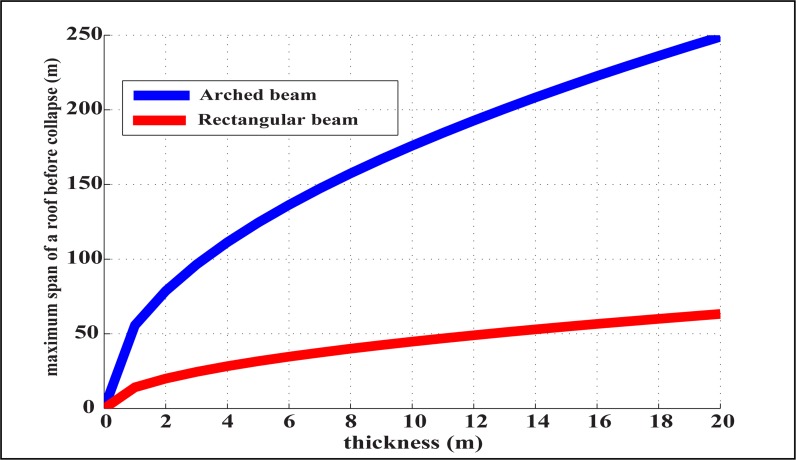
Maximum span of rectangular and arch beams as a function of beam thickness. Overall, an arched beam is more stable than a rectangular beam. For increasing beam thickness, the growth rate of stability is higher for the arched beam, as opposed to the rectangular beam.

Finally, we use (Eq 9) to find the maximum span (L) to be

$$L=2R_{a}=\sqrt{4h\sigma_{\max}\;/(3\pi -4)\rho 'g}$$ (10)

which, for *h* = 20 m, *ρ*′ = 1700 kg/m^3^, and *σ*_*max*_ = 70 MPa (maximum compressive strength of limestone), gives *L = 248*.*9m*. As expected, this is considerably larger than both the flat beam case (Fig 6) and typical values found in Florida caves. This implies, once again, that Florida caves are structurally stable.

## The Lost Buoyancy due to Bubbles

Having established that Florida caves are structurally stable, we now proceed to our proposed buoyancy reduction due to bubbles theory. Before doing so, however, it is useful to review some basic principles of bubble dynamics adopted from [8–13], where the rising velocity, shapes and stability of the bubbles are discussed.

### a) Bubble dynamics

**Fig 7:**
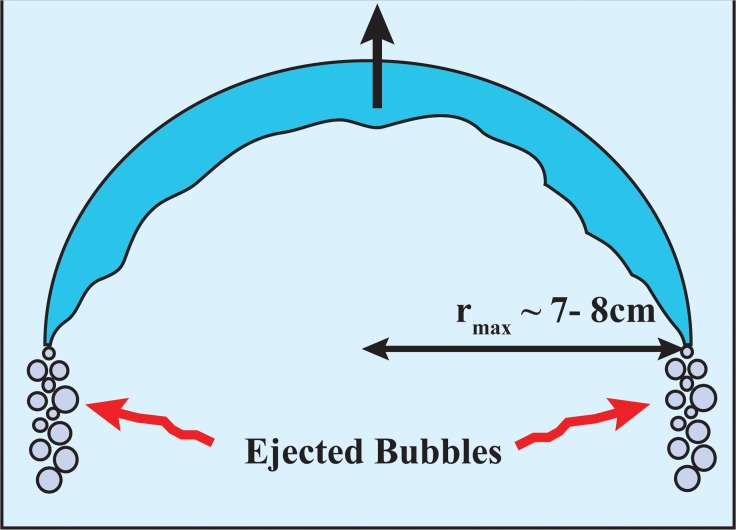
Schematic of a spherical cap-bubble rising through a liquid. As the bubble rises, the environmental pressure decreases. However, surface tension imposes a maximum bubble size (r_max_) beyond which the bubble is unstable and breaks up (~ 7–8 cm). Consequently, the bubble maintains its maximum stable size by ejecting gas around the rim as it rises.

Bubbles released by divers are usually cap bubbles (Fig 7). They rise at what is generally referred to as a “terminal” velocity of roughly 0.5 m/s (i.e., just like the balance associated with a skydiver, the driving force (buoyancy in the case of bubbles) is balanced by form-drag). For more information, the reader is referred to the outstanding and informative article of [10], and textbook of [9].

Bubbles are subject to surface-tension-controlled instability, associated with waves on their upper and lower interfaces. These interfaces can intersect, causing the bubble to break. Thus, this instability determines the maximum size of a bubble, which is approximately 7–8 centimeters. When a bubble, having reached its maximum size, rises, it attempts to accommodate for the decrease in environmental pressure by increasing its size further. Due to the above-mentioned instability, such an increase is prohibited. As a result, the bubble ejects smaller bubbles along its periphery (see Fig 7), allowing it to retain its maximal size. For other interesting aspects of bubble dynamics, the reader is referred to [14] and his discussion of static bubbles generated by dolphins.

### b) Buoyancy reduction process

Archimedes law states that the buoyancy of a submerged object is the weight of the liquid that the object displaces as it is forced into the liquid. As should be the case, it is identical to the surface integral of the vertical hydrostatic pressure exerted on the object. Hence, a rock (whose density in air, *ρ*, is 2.7 *gr/cm* ^*3*^) attached to the ceiling of a submerged cave weighs 37% less in water than in air (i.e., its in-water density is 1.7 *gr/cm* ^*3*^). When the water in which the rock is submerged contains bubbles, the buoyancy is reduced (since water with bubbles weighs less than water with no bubbles), making the effective weight of the rock larger. Taking *α* to be the fraction of the water volume occupied by bubbles (less than unity), then the relative increase of the rocks weight due to bubbles would be, *αρ*_*W /*_ *ρ*, where *ρ*_*W*_ is the water density (without bubbles).

**Fig 8:**
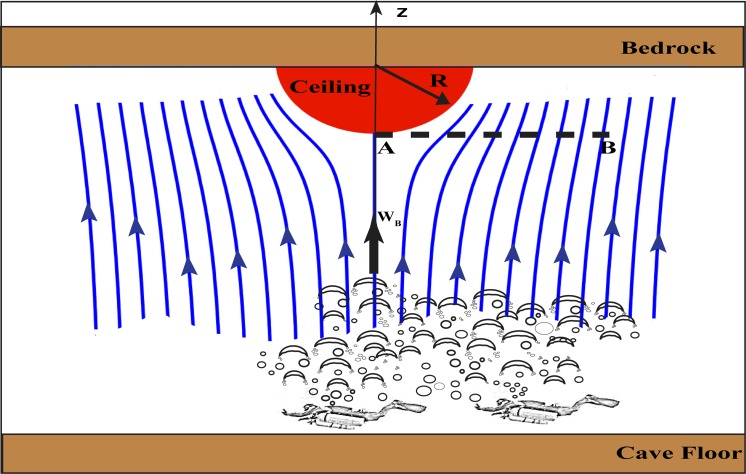
Streamline of bubbles around a semi-hemispherical ceiling rock. The radius of the ceiling rock is R. The ceiling rock is completely immersed in water. Bubbles released by the divers create a flow around the rock. Point “A” represent the stagnation point on the ceiling rock whereas “B” represents a point at the same depth where there is no ceiling rock. The bubble terminal velocity is W_B_. Because of the bubble flow, there is an excess pressure exerted on the ceiling causing a drag on the rock.

As a simple example, consider a semi-spherical rock (Fig 8) with a radius of half-a-meter (0.5 m) attached to the ceiling of a cave. The ceiling is 5 meters below the water surface in the basin into which the spring debouches and its bottom is 2 meters below its ceiling. It is 2 meters broad and has minimal flow in it. The rock weighing 706 kg in air, weighs 445 kg in water without bubbles. A cave diver lingers near the bottom of the cave, under the rock, releasing gas at a typical breathing rate of 16 liters per minute (about 0.6 cubic feet per minute). Note that these 16 liters per minute are normally measured in reference to *surface* air consumption (i.e., volume of air per minute consumed at atmospheric pressure), not the consumption of *pressurized* gas at depth. However, since the diver’s absolute lungs volume times the breathing rate (not consumption of pressurized gas) does not change with depth, it is also 16 liters at depth (though this gas is normally under higher pressure than it is at the surface). These 16 liters turn into 18 liters upon rising from the bottom to the ceiling of the cave, because the surrounding pressure reduces from an absolute pressure of 1.7 atmospheres (i.e., ambient plus atmospheric) at the bottom to 1.5 atmospheres at the top of the cave.

### c) Bubbles-rock interaction

Assuming (for the sake of argument) that the ceiling of the cave is flat so that bubbles do not escape away from the rock, we find that it will take the divers 82 minutes to displace the entire amount of water situated directly under the rock (i.e., the 1.31 m^3^). Displacing an amount equal to the rock volume (0.26 m^3^) would take 16 minutes. Without performing detailed fluid dynamics calculations, which are beyond the scope of this study, it is hard to tell what fraction of the water in the cave participates in the buoyancy reduction process. Clearly, the entire water volume of the cave does not participate in the process, nor is it merely the water directly beneath the rock that responds to the loss of buoyancy. Thus, for simplicity, we shall assume this volume to be equal to the rock volume and that the relevant water is situated immediately beneath the rock. This means *α* = 1 is achieved after 16 minutes and 5 minutes of bubbles gives us *α* = 0.3. With the latter, the rock will now weigh 533 kg, as opposed to the original 445 kg, corresponding to a 19% weight increase. For certain rocks, this amount is sufficient to cause them to fall, as our qualitative laboratory experiments will demonstrate shortly.

It is important to realize that there are actually *two* opposing bubble-induced forces acting on the rock. The first is the reduced buoyancy discussed above and the second is an opposing force directed towards the ceiling by the excess pressure, resulting from the arrest of the vertical fluid speed at point A (Fig 8). This force has not been discussed yet and will now be addressed. We refer to it as a drag-force (not lift) even though it is pointed vertically, because, by definition in fluid dynamics, drag is a force parallel to the flow whereas lift is perpendicular to the flow. To estimate this drag-force we apply Bernoulli’s principle between points A and B noting that, in the absence of vorticity, one can apply Bernoulli’s principle between any two points in the field, not only between points on the same stream line,

$$W_{B}^{2}/2+P_{B}/\rho_{W}=P_{A}/\rho_{W}$$ (11)

**Fig 9:**
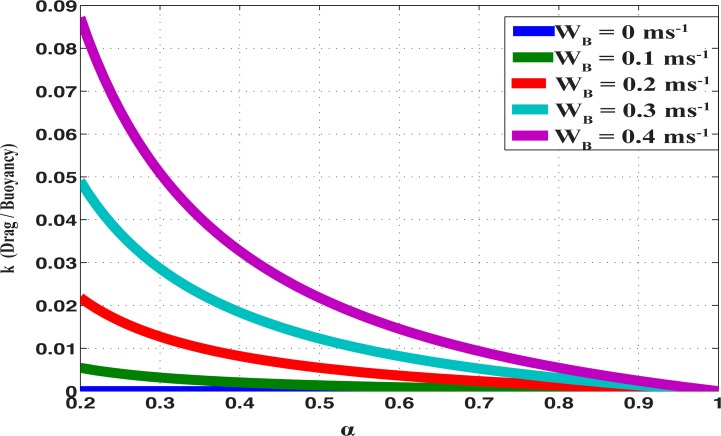
The drag / buoyancy loss ratio (k) as functions of bubble volume (α) and the vertical bubble velocity (W_B_). The ratio decreases as α increases and W_B_ decreases. However, the ratio is always small (compared to unity) for any α and W_B_, hence the drag force is negligible compared to the buoyancy loss.

Here, *P*_*B*_ the pressure at point B (Fig 8), is equal to *P*_*A* 0_, where *P*_*A* 0_ is the pressure at A without the rock present in the field. *P*_*A*_ is the pressure at point B. *W*_*B*_ is the weight of the ceiling rock in water. The excess force on the rock, due to the stagnation point at A, is at the most$\pi R^{2}W_{B}^{2}\;\rho_{W}/2$, where, *R* is the radius of the ceiling rock. Using Archimedes Law, the bubble induced buoyancy loss is *α times weight of displaced fluid = 2 πR* ^*3*^ *α*ρ_*W*_ *g /3*. This is much greater than the aforementioned excess force on the rock, as Fig 9 clearly demonstrates. The non-dimensional number (*k*) is the ratio of the excess force on the rock at point A (drag) to the bubbles induced buoyancy loss.

## Laboratory Experiments

To illustrate our ideas more vividly, we performed two sets of qualitative laboratory experiments. By “qualitative”, we mean that our experiments are intended to demonstrate a process, rather than establish “proof” of our theory or an examination of numerical values. As a result, the bubbles-to-rock length scale ratio is not comparable between the laboratory and real cave scenarios. Similarly, the bubble size used in the laboratory experiment is not realistic.

**Fig 10:**
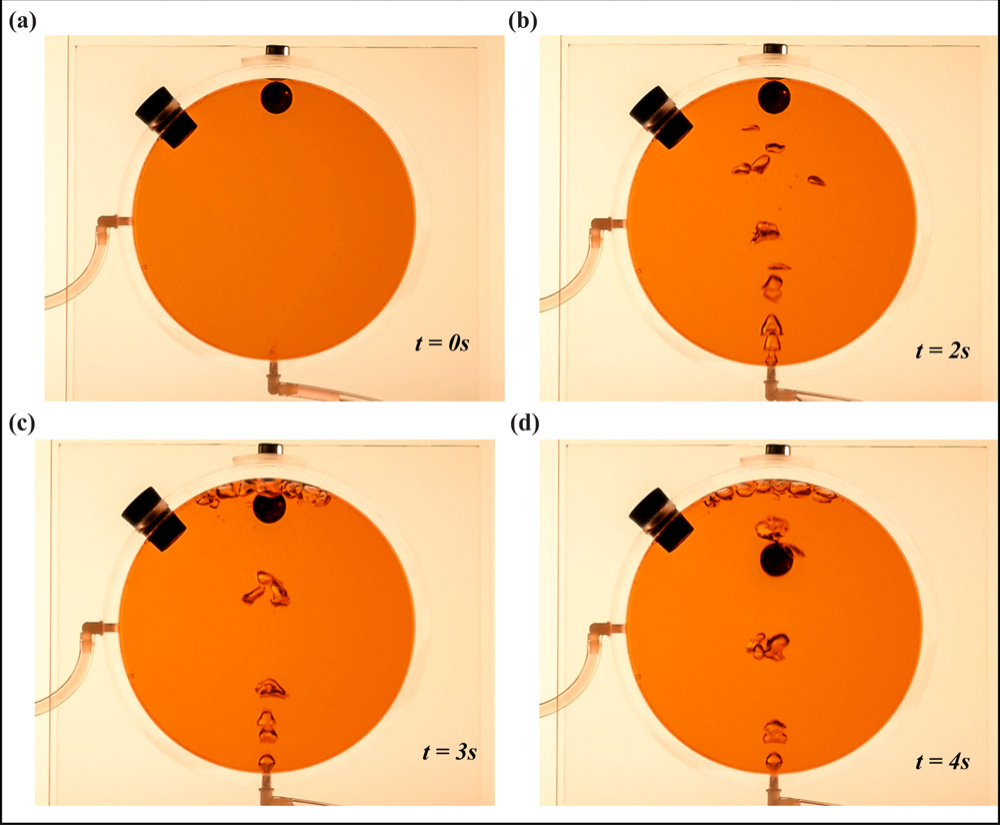
Laboratory experiment for bubble-induced cave collapse (with a concave ceiling). The apparatus is a cylindrical chamber (radius and width are 6.4 cm and 3.3 cm, respectively) filled with water (colored). The chamber is equivalent to a cave with a circular cross section. A metal ball (equivalent to a ceiling rock) is attached to the concave surface of the cylindrical chamber by a magnet. Air bubbles are released at the bottom of the chamber using a syringe. The tube on the left side of the chamber removes water displaced by the bubbles. In this demonstration, the released bubbles accumulate in the vicinity of the metal ball, due to the concave shape of the roof. (a) Experimental set up before bubble release. (b) Bubbles released at the bottom using a syringe. (c) Point at which the metal ball loses its buoyancy due to the bubbles. (d) The ball falling to the bottom of the chamber, due to gravity.

**Fig 11:**
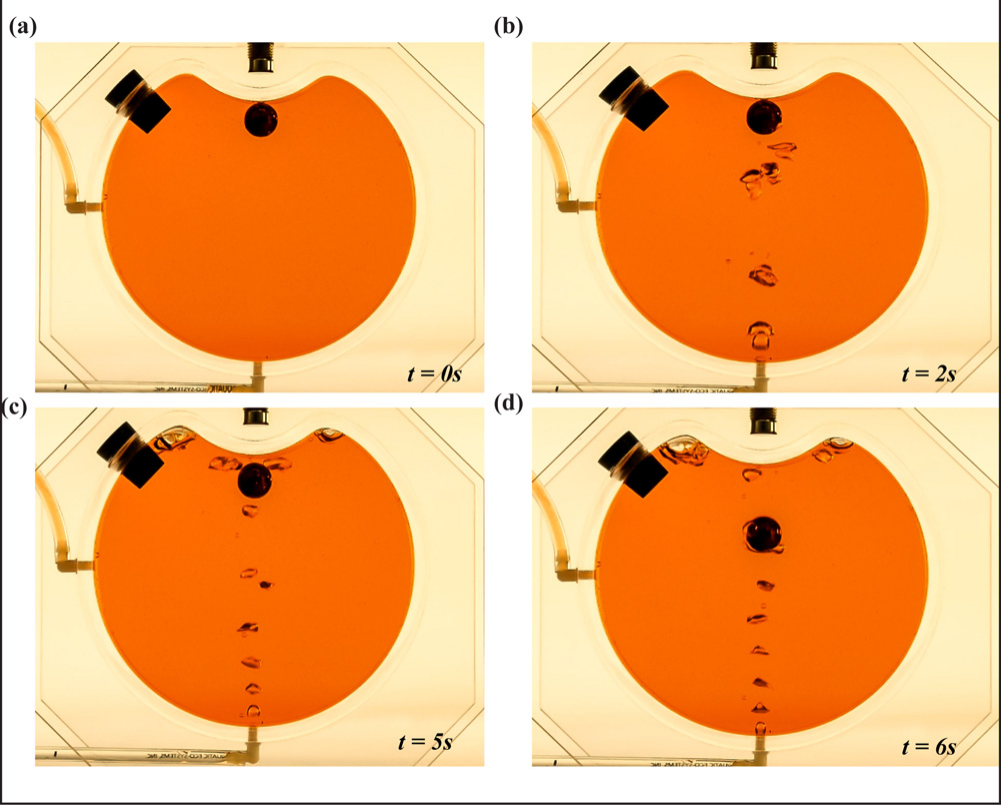
Laboratory experiment for bubble-induced cave collapse (with a convex ceiling). Now, the apparatus is a cylindrical chamber (radius and width are 6.4 cm and 3.3 cm, respectively) with a convex ceiling. It is again filled with water (colored). The chamber is equivalent to a cave with a circular cross section. Air bubbles are again released at the bottom of the chamber using a syringe. The tube on the left side of the chamber removes water displaced by the bubbles. In this demonstration, the bubbles cause a flow past the metal ball and accumulate away from the metal ball, due to the convex shape of the cave ceiling. (a) Experimental set up before bubble release. (b) Bubbles released at the bottom using a syringe. (c) Point at which the metal ball loses its buoyancy due to the bubbles. (d) The ball falling to the bottom of the chamber, due to gravity.

We constructed two small Plexiglas caves, one with a concave roof (Fig 10) and one with a convex roof (Fig 11). The radius and width of the cylindrical chamber are 6.4 cm and 3.3 cm, respectively; whereas the metal ball’s radius is approximately 0.75 cm. Bubbles are released at roughly 2-3ml/s. The concave roof, though more realistic, allows for bubbles to accumulate near the rock, which enhances the buoyancy reduction process by an amount we have not taken into account. The convex cave (Fig 11), on the other hand, allows an investigation of the process without the abovementioned bubble accumulation, despite its unrealistic configuration.

The metal “rock” was held in place by a magnet and bubbles were created by a hypodermic needle. The narrow tube on the left allowed excess fluid (created by the injection of bubbles) to be expelled out of the cave. As our calculations suggest, the rock fell down once the buoyancy was reduced beyond a critical value. This was the case regardless of the cave ceiling configuration, i.e., the site of bubble accumulation.

## Summary and Discussion

Using fluid dynamics principles, we showed that isolated ceiling rocks in submerged caves could be unstable to buoyancy perturbations due to bubbles released by divers. There is plenty of anecdotal evidence for such submerged cave collapses, however, hard evidence is difficult to come by. The only known case to be reported on in literature is that of Indian Springs in 1991. Recently, [2] proposed that this particular collapse was due to resonance in the cave. It is, however, plausible that this collapse was due to a process similar to that proposed here, i.e., rocks fell from the ceiling due to diver’s bubbles, causing a sediment slide upon impact with the marginally unstable cave floor.

The process we propose here is particularly active near cave exits, where the presence of bubbles is maximized because of their expansion due to the lower surrounding pressure. A recent case fitting this description is that of Peacock Spring (http://www.floridadiveconnection.com/florida-cave-divers-give-mother-nature-a-little-help/), though reports suggest it occurred when the park was closed (i.e., no divers were present). It is still possible, however, that divers presence earlier weakened the rock-ceiling connection, or that divers were present in the cave even though the park was closed.

Another case is that of Steve Bogaerts, who claims that a cave through which he passed collapsed behind him, forcing him to find an alternate, previously unknown, exit (see http://www.youtube.com/watch?v=wGwhLqDwmD8). In general, Yucatan caves are probably more prone to a collapse than Florida caves because they are shallower so that bubbles are subject to greater expansion as they rise from the bottom to the ceiling of the cave. Numerous broken stalactites are found on the bottom of many Yucatan caves suggesting such collapses. However, these probably occurred when the sea level was lower than it is today and the caves were dry. During those times the stalactites were not subject to any buoyancy and were thus less stable.

Finally, we also examined the possibility that material fatigue, of the kind that was responsible for air traffic accidents in the middle of the previous century, caused the collapse (through frequent generation of bubbles). It turns out that the frequency of bubble generation in caves is a few orders of magnitude smaller than that causing the collapse of windows in pressurized airplanes [15], so the likelihood of this mechanism being responsible for the limestone collapse is very small.

## Pragmatic Recommendations

Our analysis suggests that caverns (the largest part of the cave that is normally closest to the spring and usually subject to daylight) are probably the most prone to a collapse. This is because they are subject to a larger number of divers due to their larger size and relative ease of diving. They are also subject to the greatest pressure difference between their bottom and ceiling, implying the largest total volume of accumulated small and large bubbles near the ceiling. Limits on the number of divers at any one time, as well as the total time allowed in a cavern, should probably be considered and imposed in certain popular caverns. Diving in particularly fragile caverns and caves should probably be limited to re-breather diving that does not generate bubbles. Cave divers are advised to watch for rocks whose volume is roughly the same as the gas they expel while passing under them.
