# Correction: Bubble-Induced Cave Collapse

**DOI:** 10.1371/journal.pone.0127829

**Published:** 2015-05-01

**Authors:** Lakshika Girihagama, Doron Nof, Cathrine Hancock

The following information is missing from the Funding section: This study was supported by the Geophysical Fluid Dynamics Institute (GFDI) (Contribution Number 470). Both LG and CH were partially supported by GFDI.
